# Use of Antioxidants for the Neuro-Therapeutic Management of COVID-19

**DOI:** 10.3390/antiox10060971

**Published:** 2021-06-17

**Authors:** Noemí Cárdenas-Rodríguez, Cindy Bandala, América Vanoye-Carlo, Iván Ignacio-Mejía, Saúl Gómez-Manzo, Estefani Yaquelin Hernández-Cruz, José Pedraza-Chaverri, Liliana Carmona-Aparicio, Beatriz Hernández-Ochoa

**Affiliations:** 1 Laboratorio de Neurociencias, Instituto Nacional de Pediatría, Secreatría de Salud, Ciudad de México 04530, Mexico; avanoyec@pediatria.gob.mx (A.V.-C.); c_apariccio@ciencias.unam.mx (L.C.-A.); 2 Division de Neurociencias, Instituto Nacional de Rehabilitación, Secretaría de Salud, Ciudad de México 14389, Mexico; cbandala@inr.gob.mx; 3 Escuela Superior de Medicina, Instituto Politécnico Nacional, Ciudad de México 11340, Mexico; 4 Laboratorio de Medicina Traslacional, Escuela Militar de Graduados de Sanidad, SEDENA, Ciudad de México 11200, Mexico; labfisiologia.emgs@udefa.edu.mx; 5 Laboratorio de Bioquímica Genética, Instituto Nacional de Pediatría, Secretaría de Salud, Ciudad de México 04530, Mexico; saulmanzo@ciencias.unam.mx; 6 Departamento de Biología, Facultad de Química, UNAM, Ciudad de México 04150, Mexico; estefani.hernandez@quimica.unam.mx (E.Y.H.-C.); pedraza@unam.mx (J.P.-C.); 7 Laboratorio de Inmunoquímica, Hospital Infantil de México Federico Gómez, Secretaría de Salud, Ciudad de México 06720, Mexico; beatrizhb_16@comunidad.unam.mx

**Keywords:** COVID-19, SARS-CoV-2, antioxidants, oxidative stress, neurological damage, neuro-therapeutic management

## Abstract

Coronavirus Disease 2019 (COVID-19), caused by Severe Acute Respiratory Syndrome Coronavirus 2 (SARS-CoV-2), is an emergent infectious disease that has caused millions of deaths throughout the world. COVID-19 infection’s main symptoms are fever, cough, fatigue, and neurological manifestations such as headache, myalgias, anosmia, ageusia, impaired consciousness, seizures, and even neuromuscular junctions’ disorders. In addition, it is known that this disease causes a series of systemic complications such as adverse respiratory distress syndrome, cardiac injury, acute kidney injury, and liver dysfunction. Due to the neurological symptoms associated with COVID-19, damage in the central nervous system has been suggested as well as the neuroinvasive potential of SARS-CoV-2. It is known that CoV infections are associated with an inflammation process related to the imbalance of the antioxidant system; cellular changes caused by oxidative stress contribute to brain tissue damage. Although anti-COVID-19 vaccines are under development, there is no specific treatment for COVID-19 and its clinical manifestations and complications; only supportive treatments with immunomodulators, anti-vascular endothelial growth factors, modulating drugs, statins, or nutritional supplements have been used. In the present work, we analyzed the potential of antioxidants as adjuvants for the treatment of COVID-19 and specifically their possible role in preventing or decreasing the neurological manifestations and neurological complications present in the disease.

## 1. Introduction

Coronavirus Disease 2019 (COVID-19) is a highly contagious and deadly infectious disease with a broad spectrum of clinical manifestations. COVID-19 is caused by Severe Acute Respiratory Syndrome Coronavirus 2 (SARS-CoV-2). It was first identified in Wuhan, Hubei, China at the beginning of December 2019 and was declared a global pandemic in March 2020, causing around of 140,000,000 cases and up to 3,000,000 deaths until now [1].

SARS-CoV-2 belongs to the Betacoronavirus genus from the Coronavirinae subfamily within the family of Coronaviridae and Nidovirales order. SARS-CoV-2 shares homology with other coronaviruses responsible for severe acute respiratory syndromes such as SARS-CoV (~79.5% homology), which was first recognized in Guangdon, China in November 2002 and MERS-CoV (~50% homology) identified in 2012 in Jeddah, Saudi Arabia [2]. Coronaviruses are enveloped positive single-stranded RNA viruses with large genomes ranging from 8.4–12 kDa and round virions of 80–120 nm in diameter. The 5´terminal portion contains the open reading frames from viral replication proteins, while the 3´terminal encodes the structural proteins named spike (S), membrane (M), nucleocapsid (N), envelope (E), and haemagglutinin-esterase (HE) proteins. SARS-CoV-2 has a genome of 26–32 kb encoding six non-structural proteins involved in viral replication and four structural proteins [3]. The SARS-CoV-2 structure consists of a lipid bilayer where the glycoprotein type (spike) forms peplomers on the virion surface, giving it a crown-like morphology. The membrane protein spans three times the membrane surface and presents a short N-terminal ectodomain and a cytoplasmic tail, while the E protein travels twice the surface and is constituted by an N- and a C- terminal internal domain, a short ectodomain, a transmembrane domain, and a cytoplasmic tail. Some coronaviruses have a haemagglutinin esterase protein; the role of this has not been fully understood, however, in the SARS-CoV-2 genome, HE is not encoded [4].

The mutation rate of SARS-CoV-2 has been estimated to be between 0.84–1.12 × 10^−3^ substitutions per site per year [2,5]. However, transmission of the SARS-CoV-2 virus and anti-virus treatments used for COVID-19 can favor genetic variability of the virus, contributing to its load, virulence, and the variability of neuropathological findings [6]. For example, the SARS-CoV-2 variant of spike protein D614G has been the most prevalent form in COVID-19 disease and has been linked to a higher viral load in the upper respiratory tract but not to an increase in disease severity [7]. A recent study identified 5775 genome variants, including almost 3000 missense mutations, 1965 synonymous mutations, 484 mutations in non-coding regions, 142 non-coding deletions, 100 in-frame deletions, 66 non-coding insertions, 36 stop-gained changes, 11 frameshift deletions, and two in-frame insertions [2,8]. It is essential to mention this because as the pandemic is still active, more variants are being identified; this has been reported internationally with the Indian variants, whose impact on the infection rate, as well as inducing severe symptoms of this infection, are still in progress.

SARS-CoV-2 is transmitted mainly by respiratory droplets moving from one person to another [9]. For a sensitive detection of COVID-19, the collection and testing of both upper and lower respiratory samples, including sputum and bronchoalveolar lavage fluid is recommended. Several research committees have suggested that RT-PCR for COVID-19 nucleic acid detection of nasopharyngeal and oropharyngeal swab sampling and further confirmation by next-generation sequencing is the best way to diagnose COVID-19 infection [10]. The main symptoms of COVID-19 are fever, shortness of breath, cough, fatigue, headache, myalgias, anorexia, and chest pain. Other manifestations could include diarrhea, sore throat, anosmia, ageusia, hemoptysis, sputum production, rhinorrhea, nausea, vomiting, skin rash, impaired consciousness, and seizures. The presence of comorbidities during COVID-19 infection such as hypertension, diabetes, chronic respiratory disease, cardiovascular disease, cancer, or advanced age can negatively impact the prognosis of the disease [11,12,13]. The systemic complications in COVID-19 include adverse respiratory distress syndrome, cardiac injury, acute kidney injury, and liver dysfunction [14]. In addition, damage to the central nervous system (CNS) has been linked to COVID-19 infection; initial neurological characterization of COVID-19 disease in a Wuhan cohort of COVID-19 patients showed a low incidence of neurological complications such as headache, nausea, and vomiting, however, more recent studies have reported that neurological manifestations of SARS-CoV-2 infection can reach more than 35%, evidencing the neuro-invasive potential of SARS-CoV-2. Mild neurological dysfunctions such as anosmia and dysgeusia during COVID-19 are frequent, however, severe neurological disorders such as stroke and encephalopathies have also been reported, although less frequently. On the other hand, post-mortem brain studies have shown association between SARS-CoV-2 infection and pan-encephalitis and meningitis in addition to diffuse edema, gliosis with diffuse activation of microglia, and astrocytes infarctions in cortical and subcortical areas, subarachnoid and punctate hemorrhages, arteriosclerosis, hypoxic-ischemic injury, and inflammation [15]. Regardless of the lack of studies analyzing SARS-CoV-2 CNS invasion, there is information that suggests the presence of the virus in human brain tissue, such as the detection of SARS-CoV-2 RNA in the cerebrospinal fluid of infected patients [16]. In addition, other human coronaviruses have shown to be able to infect neural cells [17]. Neurological manifestations as well as abnormalities in brain imaging have been reported during infections with SARS-CoV and MERS-CoV. Moreover, particles and genomic sequences of SARS-CoV have been detected in the post-mortem brain tissue of SARS patients as well as in cerebrospinal fluid. Data showed that SARS-CoV is presented in the thalamus, brainstem, hypothalamus, and cortex but not cerebellum in both humans and animals [18]. More recently and using human brain organoids as the experimental model, it has been shown that SARS-CoV-2 can damage the choroid plexus epithelium and impair the normal function of the blood brain barrier [19]. Politi and coworkers report the follow up of brain changes during COVID-19 development in three patients [20], and Bougakov et al. suggest the infection of brain tissue by SARS-CoV-2 by axonal transport through cranial nerves according to the route: nasal cavity olfactory nerve, olfactory bulb, pyriform cortex, and brainstem; the same route that has been demonstrated during HCoV OC43 infection of brain tissue [21,22]. However, the neurotropism of SARS-CoV-2 remains in debate.

Virus, such as SARS-CoV2, infection and replication in pneumocytes causes diffuse alveolar and interstitial inflammatory exudate and alveolar gas exchange disorders [23,24]. Gas exchange disorders are also linked to hypoxia in CNS by increasing anaerobic metabolism and edema. Moreover, SARS-CoV2 infection has been related to a low level of red blood cells (RBC) observed in COVID-19 patients. Several clinical reports have documented abnormal findings in different brain areas associated with blood vessel damage that led to stroke events [25,26]. Hypoxemia reported in COVID-19 patients could be in many cases silent (happy hypoxemia). However, low levels of oxygen lead to damage in several tissues and it has been suggested that this can increase infection by up regulation of furin (via HIF-1α), a host enzyme required for cleaving the S protein of the SARS-CoV2 [27,28]. Hypoxemia has also been related to proinflammatory cytokines [29]. 

CoV infections are mainly associated with cytokine production, inflammation, and cell death, which are pathophysiological processes also related to redox imbalance or oxidative stress both in animal models and humans [30,31,32,33]. Evidence indicates that the participation of oxidative stress in the pathogenesis of COVID-19 is achieved by enhancing the production of reactive oxygen species (ROS) and causing an imbalance of the host antioxidant system. In addition, the pro-inflammatory state caused by some comorbidities has been suggested as a negative factor for COVID-19 prognosis. Respiratory hypoxia associated with COVID-19 infection could initiate a hypoxic state in the brain and thus trigger oxidative stress. It has been largely documented that hypoxia induces ROS production which are involved in inflammation and immune response. High levels of ROS are the main cause of redox imbalance, macromolecules peroxidation, and the opening of the permeability transition pores of the mitochondria, thus, cellular changes caused by oxidative stress could lead to cell death and contribute to brain tissue damage [34]. Moreover, the oxidative stress triggered by SARS-CoV-2 infections has been compared with the one involved in Parkinson’s disease and has demonstrated the activation of nuclear factor kappa B (NF-κB) [35]. It is important to consider that these pro-inflammatory processes are both central and systemic.

Currently, over 150 anti-COVID-19 vaccines are under investigation. In relation to treatment, dexamethasone and remdesivir appear to be promising medical therapies. However, to date a specific treatment for COVID-19 does not exist, and only supportive therapies are available. Until now anti-inflammatory drugs, immunomodulators, anti-vascular endothelial growth factors, modulating drugs, statins, or nutritional supplements have been studied as possible therapeutics agents [36]. The role of antioxidants as possible adjuvants in the neurological manifestations and complications of COVID-19 is reviewed in the present work.

## 2. Pathophysiological Mechanisms of SARS-CoV-2 and Its Neurological Implications

Cell entry of SARS-CoV-2 is mediated mainly by the interaction between the viral trimeric S protein and the cellular angiotensin converting enzyme 2 (ACE2) receptor [37]. The SARS-CoV-2 spike protein determines host tropism by binding to cell receptors through its receptor-binding domain (RBD) and initiates fusion and infection processes. Trimeric S protein has two functional subunits (S1 and S2). S1 binds to ACE2 receptors and induces conformational changes in S2, facilitating infection by membrane fusion. Two heptad (HR-1 and HR2) domains are present in the S2 subunit of the S protein, which play the central role in the fusion membrane during the infection process. The binding of the S protein to ACE2 through the RBD-S1 subunit allows the combination of HR1 and HR2 to form a six-helix bundle core fusion structure (6HB) and enables the proximity of the virus to the cell membrane for fusion [38,39,40].

The ACE2-receptor is a type-I transmembrane receptor with a catalytic extracellular domain, one transmembrane domain, and a cytoplasmic carboxyl domain. The extracellular portion of the ACE-2 receptor is a zinc metallopeptidase catalytic site and the spike binding domain [41]. Similarly to SARS-CoV, SARS-CoV-2 binds to ACE-2 but with higher affinity (10 to 20-fold), being more pathogenic. Viral S-protein priming by cellular transmembrane protease serine 2 (TMPRSS2) exposes its binding S1 domain and fusion S2 domain. The S1 binding to the ACE-2 receptor induces its internalization by upregulation of the ADAM metallopeptidase domain 17 (ADAM 17) activity which cleaves ACE2 from the cell surface. S2 domain exposure starts the viral fusion process to gain entry into cells [39,42] and release the viral genome into the cytoplasm where host ribosomes translate a polypeptide chain (~800 KDa) that is auto-proteolytically cleaved by two proteases: papain like protease (PLpro) and 3-chyomotrypsin like protease (3CLpro), also called the main protease (Mpro), which are encoded in the viral genome and generate the no-structural proteins required for viral replication [43].

The main ACE-2 function is associated with the cleavage of the renin–angiotensin–aldosterone system (RAAS) peptides and is a regulatory mechanism opposed to the effects of angiotensin II generated by ACE. RAAS is a neurohormonal regulatory system involved in blood pressure and electrolyte homeostasis. Angiotensinogen is produced by the liver and cleaved into angiotensin I (Ang I). ACE catalyzes Ang I conversion to angiotensin II (Ang II), which is the main RAAS metabolite and binds to angiotensin II type 1 receptors. Ang II actions include vasoconstriction, renal sodium reabsorption and potassium excretion, aldosterone synthesis, blood pressure elevation, and inflammatory and pro-fibrotic signaling. ACE-2 cleaves Ang II to Ang (1–7) and exerts vasodilatation, anti-inflammation, and anti-fibrotic effects by Mas receptor system activation. RAAS activation effects depend on the tissue ACE/ACE2 balance, which could be affected by several factors [44,45,46].

ACE and ACE2 expression have been reported in almost all tissues such as vascular endothelia, lungs, brain, intestine, colon, heart, testis, pancreas, eye, thyroid, adipose tissue, gallbladder, and kidneys [47,48]. Interaction between SARS-CoV-2 and the ACE-2 receptor could affect the ACE/ACE2 balance, causing high levels of Ang II and activating Ang II/AT1R signaling. Tissues expressing elevated ACE2 are potential targets for SAR-CoV-2 infection, such as intestine, kidney, testis, gallbladder, and heart [48]. Because of the virus transmission mechanism, the lung is the main target organ for SARS-CoV-2. After entrance of SARS-CoV-2 into pneumocytes vascular permeability and inflammation have been reported, which has been related to ACE-2 downregulation. In addition, studies have demonstrated that Ang II level has a positive correlation with viral load and lung injury. In vitro studies have demonstrated that AT1R activation by Ang II can induce apoptotic death of lung epithelial cells. It has been shown that Ang II induces endothelial damage by cyclooxygenase (COX-2) activation, which in turn generates vasoactive prostaglandins and reactive oxygen species (ROS) [49,50]. The excessive production of ROS can then over activate AngII/AT1R/nicotinamide adenine dinucleotide phosphate (NADPH) oxidase axis and subsequently induce apoptosis by mitochondrial injury [51]. Release of cytochrome C, activation of caspase 3, and p38 mitogen activated protein kinase (MAPK)/Jun N-terminal kinase (JNK) cascade activation have been related to elevated ROS levels. Moreover, the entry of SARS-CoV-2 can cause destruction of lung cells by activating a local immune response mediated by macrophages and monocytes; these cells release cytokines such as interleukin-6 (IL-6), interferon-γ (IFN-γ), monocyte chemoattractant protein-1 (MCP-1) interferon-γ-inducible protein-10 (IP-10), or tumor necrosis factor (TNF) into the blood of patients, thus being indicators of T-cells activation. Other inflammatory pathways activated by Ang II involve the transcriptional nuclear factor NF-kB and the expression of proinflammatory cytokines such as IL-6, IL-1β, and TNFα [52]. In COVID-19 patients, an excessive cytokine release has been documented, which induces an increase in leukocyte recruitment to different body organs leading to multi-organ failure and could result in acute heart injury or acute renal injury. This phenomenon is called cytokine storm syndrome, it also occurs in other viral diseases such as SARS, MERS, and influenza. The result of the ACE2 protection loss is a hyperinflammatory state which can be seen the late phase of COVID-19 [53].

Previous studies on SARS-CoV and MERS-CoV have shown that coronaviruses are able to infect CNS cells in the brainstem, which suggests than infection of this brain region during COVID-19 could compromise respiratory and cardiovascular function [54]. However, the SARS-CoV receptor is different to the MERS-CoV one, which has been reported use Dipeptidyl peptidase 4 (DPP4) to gain access into different tissues, among them the cerebral cortex [55,56]. In relation to the entry of SARS-CoV-2 in CNS, it has been shown that ACE2 expression is highest in the amygdala, pons, and medulla oblongata and then also related to the susceptibility of the subject to respiratory distress [57]. It has been proposed that the SARS-CoV-2 neurovirulence could be related to the degree of expression of the ACE2 receptor in the regions of the CNS [58]

Employing an animal model demonstrated that SARS-CoV accessed brain tissue through the olfactory bulb, data that can be related to anosmia generated during COVID-19 [59]. Moreover, it has been suggested that ACE2 could mediate SARS-CoV-2 neurotropism since it is expressed in neurons, astrocytes, and oligodendrocytes, mainly in the substantia nigra, ventricles, middle temporal gyrus, posterior cingulate cortex, and olfactory bulb as well as endothelial cells. In humans, ACE2 has a relatively high expression in the middle temporal gyrus and posterior cingulate cortex but is low in the hippocampus. Its expression has been showed also in the tractus solitarius nucleus, paraventricular nucleus, and rostral ventrolateral medulla, which are regions implicated in cardiovascular regulation [48,60]. Importantly, it has been demonstrated that SARS-CoV-2 can disrupt the brain blood barrier and gain access to brain tissue [19].

Currently, the mechanisms by which SARS-CoV-2 can disturb neurological functions are not known. However, some hypotheses have been proposed. The first one states that the neurological manifestations rise due to direct neurotropic action of the virus causing encephalitis or meningitis; the virus enters the CNS. Virus entry could be from direct blood circulation infecting vascular endothelium, through nasal cells invading the olfactory epithelium along the nerve to the olfactory bulb, or by leukocyte migration across the BBB and neuronal pathways to the Virchow-Robin space surrounding arterioles and venules, and into the lymphatic systems and receptors [47,61]. The other mechanism that has been proposed is the parainfectious disease mechanism, which results in immune-mediated nerve disturbance such as Guillain–Barre syndrome or Miller Fisher syndrome [62]. In addition, it has been shown that the existence of some risk factors predisposes patients with COVID-19 to neurological complications. Among the most common are older age and the presence of comorbidities, particularly hypertension and smoking since stimulation of the nicotinic acetylcholine (nACh) receptor could increase ACE2 expression in neurons [63]. However, further research is needed in order to clear the mechanism by which SARS-CoV-2 affects brain tissue due to the low expression of ACE2 in human brain [48]

Regardless of the kind of interaction between SARS-CoV-2 and brain tissue, the effects on the CNS are present during COVID-19 disease. Among the most common are smell impairment with normal nasal mucosa and normal imagining of olfactory bulbs; cerebrovascular disease, mainly ischemic events in small and large vessels. Stiff neck, confusion changes in mental status, or seizures have also been reported. Related to peripheral nerves, injury to cranial nerves and muscles has been associated with facial weakness, difficulty breathing, and trouble standing or walking [64]. Cytokine increased production (IL-6, IL-8, IL-10, I, and TNF-α) and microglial activation has been observed in post-mortem brain tissue, and T-cell infiltration has been described in post-mortem brain tissue through mild perivascular infiltration [65], oxidative stress triggered by hypoxia, hypercoagulation and thrombosis [66], gut microbiome dysbiosis [67], unfolded protein response, and accumulation of misfolded proteins such as amyloid-beta/tau/alpha-synuclein [68] and neurological autoimmune response [62], which are the main events that could explain the neurological symptoms during COVID-19.

COVID-19 infection produces mild neurologic manifestations such as headache and loss of smell. Globally, asthenia, myalgia, headache, anosmia, and ageusia are the most common symptoms, followed by encephalopathy, stroke, and seizures [3]. Near to 36% of COVID-19 patients exhibit neurological symptoms, including both central and peripheral signs. The hypercoagulation state observed during COVID-19 disease also affects CNS integrity and damage to the brain vasculature has been observed in 2% of patients [69,70,71,72].

Encephalopathy is considered the most common CNS complication of COVID-19 [73]; about 50% of the hospitalized COVID-19 cases develop it [74]. In addition, age and pre-existent cognitive impairment, several comorbidities, malnutrition, concomitant infections, metabolic disorders, liver, vascular, and kidney dysfunctions, and sepsis are considered risk factors for neurological damage in COVID-19 patients [6]. Anatomopathological findings in the post-mortem brain tissue of COVID-19 patients are the presence of neuroinflammation with encephalitis, hemorrhagic lesions, infarctions, thrombosis, acute cerebral and cerebellar hypoxia-related lesions, reactive gliosis, astrocytosis, and microglia activation, showing a relationship between SARS-CoV-2 and central nervous system sequelae [75]. Table 1 shows the brain damage or neurological manifestations induced by COVID-19 infection reported in clinical cases.

## 3. Pharmacological Treatment for Patients with Neurological or Psychiatric Manifestations Associated with COVID-19

General treatment of COVID-19 patients has been directed toward reducing symptomatic manifestations and maintaining vital functions, controlling comorbidities, and preventing secondary infections. World Health Organization (WHO) recommendations allow the use of some drugs with antiviral capacity for COVID-19 treatment: the antivirals lopinavir/ritonavir (Kaletra^®^) affect proteolysis during SARS-CoV-2 replication cycle [145]; remdesivir is an adenosine analog able to incorporate into nascent viral RNA chains which results in premature termination [146]; and chloroquine and hydroxychloroquine, which have been traditionally used as antimalarial agents inhibit viral entry, uncoating, assembly, and budding processes [147]. In addition, other agents have been used or proposed to diminish the damage induced by COVID-19 such as: tocilizumab, an immunosuppressive agent IL-6 receptor antagonist [148]; antibiotics such as bafilomycin A1 [149] and Azithromycin, in combination with hydroxychloroquine [150]; anti-inflammatory agents as glucocorticoids [20]; JAK inhibitors such as baricitinib [151]; and micronutrients as vitamins [152].

Treatment and clinical management of patients with neurological manifestations in cases of severe or critical illness include mechanical ventilation and intensive care unit for life support. Vascular and inflammatory complications are the most common neurological effects during COVID-19 disease, so anticoagulants with low-molecular-weight are administered to the patients with coagulopathies and thromboprophylaxis to prevent stroke (nadroparin 2850–5700 IU sc per day, 5700 IU per day with body weight > 100 kg, or nadroparin 5700 IU sc every 12 h) [153], plus reperfusion therapy with alteplase (0.9 mg/kg) have been successfully used. In advanced COVID-19 disease, when anticoagulation with systemic heparin has failed, rescue therapy with tissue plasminogen activator (tPA) can be used to restore microvascular patency [121,154,155]. Due to the capacity of the antimalarial drug mefloquine to cross the BBB, the use of chloroquine and hydroxychloroquine has been proposed as candidates to treat neuroinflammation induced by COVID-19 [156].

Psychiatric disorders reported that for the management of anxiety and agitation in COVID-19 patients, the following drugs have been recommended and used successfully: in patients over 18 years of age with swallowing ability lorazepam 0.5–1 mg orally four times a day (maximum 4 mg in 24 h) [157,158], in elderly or debilitated patients the dose should be reduced to 0.25–0.5 mg (maximum 2 mg in 24 h); for anxiety or agitation with inability to swallow it is recommended to use midazolam 2.5–5 mg every 2–4 h as needed, and if frequently needed an infusion of 10 mg over 24 h. For delirium manifestations in patients with swallowing capacity, it is recommended to use 0.5–1 mg of haloperidol at night and every 2 h when needed (maximum 10 mg daily, or 5 mg daily in elderly patients) and this can be administered subcutaneously; a higher initial oral dose is recommended (1.5–3 mg) if the patient is very distressed or is causing immediate danger to others, as well as the addition of benzodiazepines such as lorazepam or midazolam [158] if the patient remains agitated. Olanzapine (10 mg per day) has also been successfully used in the treatment of severe COVID-19 and schizophrenia (tension, panic, anxiety, aggression, and paranoia); midazolam, diazepam, and dexmedetomidine were administered to relieve anxiety and aid sleep [158,159]. In addition, agomelatine use (36 mg/day and 72 mg/day orally in four doses) has been reported to improve the capacity for sleep [160]. Intravenous valproic acid (titrated to 1250 mg per day) has been used in patients with COVID-19 for the management of agitation and hyperactive delirium symptoms and to facilitate tapering of multiple other delirium-sedative medications [161]. Some authors recommend candidate drugs to treat delirium in COVID-19 patients, such as melatonin, dexmedetomidine, clonidine, α-2 agonists, and guanfacine [162,163]. The treatment protocols used in clinical cases of neurological or psychiatric manifestations induced by COVID-19 are summarized in Table 2.

## 4. Role of Oxidative Stress and Antioxidant System in Patients with COVID-19

Reactive oxygen species (ROS) are derived from molecular oxygen but are more reactive than oxygen itself. The term ROS includes free radicals and non-radicals [165]. For example, ozone (O_3_), singlet oxygen (^1^O_2_), superoxide anion (O_2_^•−^), hydrogen peroxide (H_2_O_2_), and hydroxyl radical (^•^OH) are considered ROS. ROS play an essential role in viral infections by inducing innate immune responses due to the opening of inter-endothelial junctions that allow the migration of inflammatory cells through the endothelial barrier [166,167]. The recruitment of inflammatory cells at the site of infection causes excessive ROS production, which is considered essential for the genesis and progression of inflammatory diseases [168,169]. Furthermore, the increase in ROS levels has been shown to stimulate the severity of viral infections due to their participation in inflammatory processes and the release and dissemination of virions [147]. Several respiratory viruses, including respiratory syncytial virus (RSV), human metapneumovirus (hMPV), MERS, SARS-CoV, and influenza increase ROS formation as a result of increased recruitment of inflammatory cells at the site of infection [170]. In these viral infections, the reduction of antioxidant enzymes expression and/or activity leads to a redox imbalance and consequent oxidative cell damage [170]. Similarly, during viral infection caused by SARS-CoV-2, increased oxidative stress has been proposed due to more significant migration of neutrophils to the infected area [171]. There is evidence that patients with severe COVID-19 present an increase in neutrophils and a decrease in lymphocytes levels, which could be an important factor in the severity of the disease [172,173,174,175]. The reduction of lymphocytes, particularly T cells, has also been related to oxidative stress. In a pro-oxidant environment, the essential regulatory proteins in T cells (such as cofilin or L-plastin) are oxidized, which could lead to the hypo-responsivity of these cells and even their death [156]. The ROS-mediated activation of transforming growth factor beta 1 (TGF-β1) promoter during SARS-CoV infection has been documented. TGF-β is a potent immunosuppressive cytokine acting on T cells and could contribute to the decrease of lymphocytes in COVID-19 [176,177].

Another central mechanism that could contribute to ROS formation (including O_2_^•−^ and H_2_O_2_) in COVID-19 patients is the activation of the enzyme nicotinamide adenine dinucleotide phosphate (NADPH) oxidase (NOX). NOX catalyzes the transfer of electrons from NADPH to molecular oxygen, producing O_2_^•−^, and subsequently O_2_^•−^ is transformed to H_2_O_2_ by the action of superoxide dismutase (SOD) [178]. There are seven different isoforms of NOX (NOX1–5 and DUOX1–2) with regulation and specific subcellular locations [179]. Isoform 2 (NOX-2) has been found to be overexpressed in hospitalized patients with COVID-19 and has been associated with increased oxidative stress [180].

NOX activation is regulated by the binding of angiotensin II (Ang II) to angiotensin type 1 (AT1R) [181]. The binding of SARS-CoV-2 to ACE2 causes the virus to enter cells and, in turn, reduces the bioavailability of ACE2 [182]. The reduction in the bioavailability of ACE2 makes Ang II interact with AT1R, with the subsequent activation of NOX and induction of oxidative stress and inflammatory responses [183]. ROS generated by this route could be related to an increase in viral load [184] due to the oxidation of cysteine residues in the peptidase domain of ACE2 receptors and in the carboxy-terminal receptor-binding domain (RBD) of SARS-CoV-2 peak proteins, maintaining them in oxidized forms (disulfide); the oxidation of these thiols causes an increase in the affinity of SARS-CoV-2 proteins for its ACE2 receptor, causing more viruses to enter cells [185,186]. Liu et al. [172] found elevated levels of angiotensin II in patients infected with SARS-CoV-2 that were directly proportional to the viral load and lung damage observed. Additionally, NOX activation reduces the bioavailability of nitric oxide (NO), leading to vasoconstriction, inflammation, redox imbalance, and endothelial dysfunction [172,187] in such a way that the classic RAAS, particularly the ACE2-Ang-(1–7) axis, becomes a powerful pro-oxidant system in COVID-19. Interestingly, NOX can also be activated by the release of TNF-α during the pro-inflammatory cytokine storm, contributing to local oxidative stress and endothelial dysfunction [188,189]. TNF-α-induced ROS production could also contribute to the spread of COVID-19 symptoms to distant tissues such as the brain [190].

Oxidative stress in COVID-19 has also been linked to the release of iron into the bloodstream. SARS-CoV-2 attacks the heme groups of hemoglobin in red blood cells, producing the release of free Fe (III) ions into the bloodstream, which, through the Fenton and Haber–Weiss reactions, increases the ROS levels [191]. In addition, excess ROS causes the formation of methemoglobin, hemoglobin whose heme group has iron in the ferric state, Fe (III) (that is, oxidized). This type of hemoglobin cannot bind dioxygen, resulting in less efficient oxygen transport [171]. The effect of oxidative stress on red blood cells contributes to the hypoxic respiratory failure seen in the most severe cases of COVID-19 [151].

Mitochondria are the primary sources of ROS production in cells; therefore, mitochondrial dysfunction also plays an essential role in the oxidative stress observed in SARS-CoV-2 viral infection. Recent studies have hypothesized that the cytosolic ROS produced by NOX could trigger the opening of the adenosine triphosphate (ATP)-sensitive mitochondrial potassium channel (mitoK ATP) and the activation of the permeability transition pore (mPTP), causing the depolarization of the mitochondrial membrane and a burst of mitochondrial ROS production and the subsequent mitochondrial dysfunction [181]. Mitochondrial dysfunction has been linked to inflammation; this relationship occurs in both directions. Inflammatory mediators and immune sentinels trigger intracellular cascades that alter mitochondrial metabolism [192]. For example, TNF-α and interleukin (IL)-6 impair mitochondrial oxidative phosphorylation and coupled ATP production and trigger the production of mitochondrial ROS in the cell, leading to mitochondrial dysfunction, which has been found expressed in patients with COVID-19 [193,194]. Several studies have shown the impact of dysfunctional mitochondria on the immune response; a recent study revealed that human alveolar epithelial cells with dysfunctional mitochondria showed increased production of pro-inflammatory cytokines (CXCL-8, IL-6, CCL20, CCL3, CCL4, and IL-12), all of them increased in COVID-19 [195,196]. In the brain, hypoxia causes bioenergetic dysfunction of brain cells, also known as mitochondrial dysfunction. When a virus proliferates in lung tissue cells, it causes diffuse alveolar and interstitial inflammatory exudation, edema, and the formation of transparent membranes. This, in turn, leads to alveolar gas exchange disorders that cause hypoxia in the CNS, leading to mitochondrial dysfunction of brain cells [197].

As mentioned above, antioxidant systems are also affected by viral infections. Komaravelli and Casola [170] have associated respiratory viral infections with the inhibition of nuclear erythroid factor 2-related factor 2 (Nrf2) and activation of NF-κB, phenomena that incline the balance to inflammation and oxidative damage during these infections [170]. In a study conducted on lung biopsies from patients with COVID-19, the Nrf2 pathway was found to be suppressed, while pharmacological inductors of Nrf2 have been observed to inhibit SARS-CoV-2 replication and the inflammatory response [198]. On the other hand, it is known that Nrf2 deficiency increases ACE2 availability, while activation produces the opposite, suggesting that Nrf2 activation in COVID-19 patients could reduce ACE2 availability for entry of SARS-CoV-2 in the cell [199]. However, it must be considered that Ang II would be overexpressed and, therefore, could contribute to the increase in oxidative stress, as previously explained. Nrf2 is a transcription factor whose target genes include those of proteins involved in cellular redox homeostasis, detoxification, macromolecular damage repair, and metabolic balance [200]; therefore, a decrease in Nrf2 could be related to the decrease in the enzymes that protect against oxidative stress including thioredoxin, thioredoxin reductase, peroxiredoxin, and those involved in glutathione (GSH) synthesis, among others.

**Figure 1:**
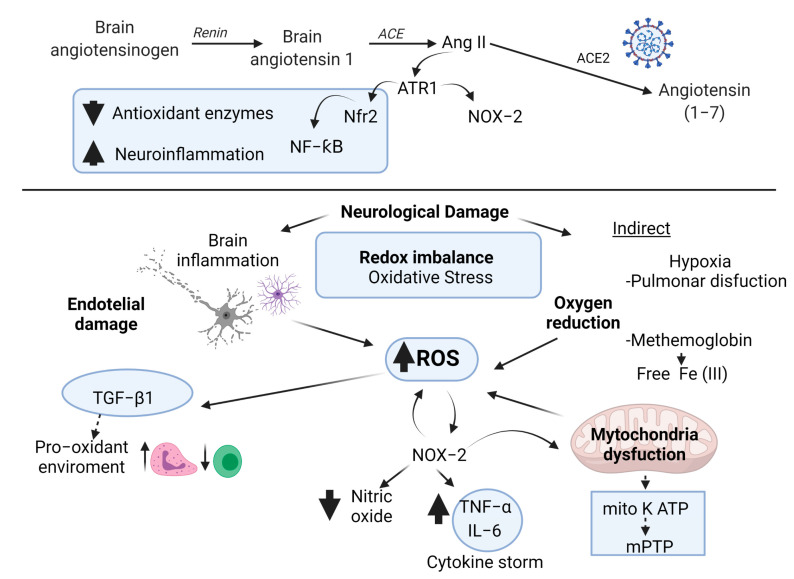
Possible mechanisms of neuronal damage related to redox imbalance in COVID-19. The recruitment of inflammatory cells increases ROS production. ROS levels have been shown to stimulate inflammatory processes and the release and dissemination of virions. An increase in neutrophils and a decrease in lymphocytes (T cells) has also been related to pro-oxidant environment mediated activation of TGF-β1. Another major mechanism that could be contributing to the ROS formation is the activation of the NOX-2. NOX-2 activation is regulated by the binding of angiotensin II (Ang II) to angiotensin type 1 (AT1R). The binding of SARS-CoV-2 to ACE2 causes the virus to enter cells and, in turn, reduces the bioavailability of ACE2. The reduction in the bioavailability of ACE2 makes Ang II interact with AT1R, with the subsequent activation of NOX and the induction of oxidative stress and inflammatory responses. NOX activation reduces the bioavailability of nitric oxide. NOX can also be activated by the release of TNF-α during the pro-inflammatory cytokine storm. SARS-CoV-2 attacks the heme groups of hemoglobin in red blood cells, producing the release of free Fe (III) ions into the bloodstream which, through the Fenton and Haber–Weiss reactions, increases the ROS levels. In addition, excess ROS causes the formation of methemoglobin, resulting in less efficient oxygen transport. In the brain, hypoxia causes bioenergetic dysfunction of brain cells, also known as mitochondrial dysfunction. Cytosolic ROS produced by NOX could trigger the opening of the adenosine triphosphate (ATP)-sensitive mitochondrial potassium channel (mitoK ATP) and the activation of the permeability transition pore (mPTP) causing the depolarization of the mitochondrial membrane and a burst of mitochondrial ROS production and the subsequent mitochondrial dysfunction. TNF-α and interleukin (IL)-6 impair mitochondrial oxidative phosphorylation and coupled ATP production and trigger the production of mitochondrial ROS in the cell, leading to mitochondrial dysfunction. Respiratory viral infections have been associated with inhibition of nuclear erythroid factor 2-related factor 2 (Nrf2) and activation of NF-κB. The decrease in Nrf has been related to a detriment in the production of antioxidant enzymes.

It has been proposed that endogenous GSH deficiency could be one of the causes of severe symptoms in COVID-19 patients. The decrease in GSH and the increase in ROS were found to have a strong correlation with the worsening of symptoms and the slowest recovery times [201]. Furthermore, GSH deficiency causes an alteration in the genes that synthesize vitamin D, resulting in a vitamin D deficiency and an increase in oxidative stress [201]. Likewise, it is known that GSH concentrations are decreased in the elderly [202], which could explain their susceptibility to the severity of COVID-19. In addition, in a study with elderly patients infected with the SARS-CoV-2 virus, a link was observed between the decreased expression of the antioxidant enzyme superoxide dismutase 3 (SOD3) and the severity of the disease [203]. It has also been proposed that GSH deficiency in a COVID-19 patient may be related to the increased NOX activity due to NOX using the coenzyme NADPH to carry out its reaction, reducing the concentration of free NADPH that is needed to regenerate GSH [204]. In Figure 1, we propose the mechanisms of neuronal damage and its relation to oxidative stress in COVID-19.

## 5. Mechanisms of Antioxidants Compounds against SARS-CoV-2 in COVID-19

The antioxidant activity of different compounds used in Chinese herbal medicine has been well documented and recently, some of them demonstrated their antiviral activity against SARS-CoV-2 [4,204]. Flavonoids are secondary plant phenolics and their bioactivity originates from their antioxidant and chelating capacity. Its chemical structure possesses a flavan nucleus and the number, position, and types of substitutions modify radical scavenging and chelating activity.

The antiviral effect of flavonoids and other antioxidant molecules against SARS-CoV-2 has been explored by means of molecular docking studies. The binding interactions of antioxidant compounds with S protein RBD and other SARS-CoV-2 proteins involved in COVID-19 infection have been studied. According to recent studies compounds like quercetin, withanolides, anaferine, ashwagandhanolide, nefamostat, hinokiflavone and robustaflavone showed high affinity for the S2 domain of the spike protein of SARS-CoV-2 [205,206]. These compounds interact with SARS-CoV-2 through its aromatic amino acid residues through non-covalent bonds (H-bonds) with the SARS-CoV-2 S2 protein. Moreover, hinokiflavone and robustaflavone interacted strongly with the residues of heptad repeat 1 and 2 regions of S2 proteins of SARS-CoV-2, inhibiting the fusion between the virus and target cell membranes [205]. Kamferol, curcumin, pterostilbene, and hydroxychloroquine have also been reported to interact with the C-terminal of the S1 domain and fisetin, quercetin, isorhamnetin, genistein, luteolin, resveratrol, and apigenin interact with the S2 domain of the SARS-CoV-2 spike protein [207].

Su and coworkers showed that baicalin and baicalein inhibited SARS-CoV-2 [208]. These compounds act as non-covalent, non-peptidomimetic inhibitors of SARS-CoV-2 3CLpro (main protease, Mpro). Baicalein works as a shield in front of two catalytic dyads and with high efficiency in the substrate-binding site at the surface of the protease [208]. Baicalin is also an ACE2 inhibitor [209]. Curcumin, quercetin, chrysin, kaempferol, luteolin melatonin, capsaicin, sesamin, cyanidins, demethoxycurcumin, epigallocatequin, hesperidin, myricitrin, puerarin, scutellarin, ursolic acid, glabiridin, apiin, rhoifolin, glycyrrhizin, vitexin, rutin, theaflavin-3-O-gallate, oolonghomobisflavan-A, bonducellpin D, caesalmin B, 5,7-dimethoxyflavanone-4′-*O*-β-*d*-glucopyranoside, lupinifolin, pinocembrin 7-O-rutinoside methide quinone celastrol, andrographolide theasinensin-D, ebselen, galangin, ellagic acid, and coumarin analogues also inhibit SARS-Cov-2 replication. Those compounds interact with SARS-CoV-2 3CLpro in the binding pocket by hydrogen bonds, hydrophilic, and hydrophobic interactions [210,211,212,213,214,215,216,217,218,219,220,221,222].

Theaflavins, neohesperidin, naringenin, echinacoside, and salvianolic acid inhibited RNA-dependent RNA polymerase activity, blocking the active site of the groove (an active site for the polymerization of RNA but distal from the active site) through hydrophobic and hydrogen interactions [223,224]. Epigallocatechin gallate, theaflavin, and rutin digallate also show interactions with SARS-CoV-2 3CLpro, SARS-CoV-2 spike protein, and ACE2 receptor through a combination of hydrogen bonding, van der Waals and other hydrophobic interactions [225,226]. Utomo and coworkers showed that hesperidin, curcumin, brazilin, and galangin show anti-SARS-CoV-2 activities because of their high affinity for the S protein RBD, PD-ACE2, and SARS-CoV-2 protease [227]. Additionally, asparosides, shatavarins, and racemoside-A also interact with S protein RBD by hydrogen bonds [228].

Compounds such as flavonoids, iridoids, terpenes, diterpenes, and lignans were also reported as promising anti-SARS-CoV-2 treatments through interaction with TMPRSS2 through van der Waals and hydrogen bonds [229,230]. Ursonic acid, asparosides, racemoside-A, and rutin also show interaction with non-structural protein (Nsp)-15 (an endoribonuclease essential for the virus life cycle) of SARS-CoV-2 via hydrogen bonds [228,231]. It has been shown that nigellidine interacts with the N-protein, and Nsp2, which has been suggested as disrupting the host cell environment by interacting with prohibitin host proteins (PHB and PHB2), is known to play roles in cell cycle progression, cell migration, cellular differentiation, apoptosis, and mitochondrial biogenesis. Nigellidine also binds to SARS-CoV-2 3CLpro and SARS-CoV-2 spike protein through diverse interactions and blocks the inflammatory markers IL1 and IL6 [232]. Additionally, it was shown that caffeic acid, ferulic acid anthrarufin, aloe-emodin, alizarine, dantron, and emodin interact with three active sites of RNA binding domains of nucleocapsid phosphoprotein of COVID-19 through hydrogen bonds and hydrophobic groups [233,234]. Finally, synthetic antioxidant compound derivatives of the pyrimidine and piperizine ring framework, polyhydroxy-1,3,4-oxadiazole, triazole, hesperidin, resveratrol derivatives and derivatives of natural products such as terpenoids and polyphenolic compounds have also shown interactions with SARS-CoV-2 3CLpro and RNA-dependent RNA polymerase activity, principally by hydrogen bonds, hydrophobic groups and van der Waals forces [222,235,236,237,238].

**Figure 2:**
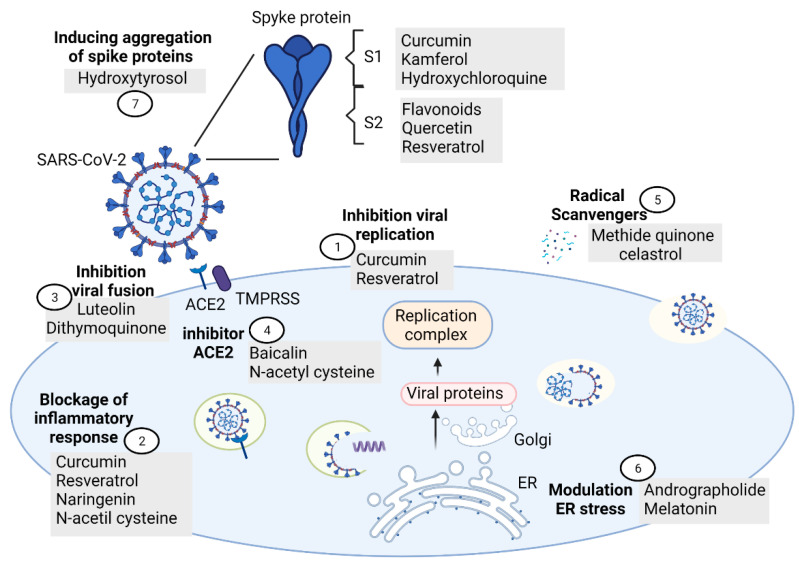
Mechanisms of antioxidant compounds that may possibly prevent and diminish neurological damage in patients with COVID-19. (1) Inhibition of the viral replication, (2) blockage of the inflammatory response, (3) inhibition of SARS-CoV-2 fusion/entry by interaction with SARS-CoV-2:ACE-2 interface, (4) inhibition of ACE2, (5) radical scavengers, (6) modulation of endoplasmic reticulum stress, and (7) inducing aggregation of SARS-CoV-2 spike proteins and disruption of the viral genoma.

Other antiviral mechanisms proposed by some authors against SARS-CoV-2 observed in antioxidant compounds are: (a) inhibition of the viral replication (i.e., curcumin and resveratrol) [239,240], (b) blockage of the inflammatory response (i.e., resveratrol, curcumin, naringenin, and N-acetil cysteine) [207,239,240,241], (c) inhibition of SARS-CoV-2 fusion/entry by interaction with SARS-CoV-2:ACE-2 interface (luteolin and dithymoquinone) [242,243], (d) as radical scavengers (i.e., methide quinone celastrol as superoxide radical scavenger or hydrogen as hydroxyl radical scavenger) [214,244], (e) inhibition of ACE activity (N-acetyl cysteine) [245], (f) modulation of endoplasmic reticulum stress (andrographolide and melatonin) [246], (g) activation of Nrf2 pathway (antioxidant mixture PB125^®^) [247], (h) antithromboembolics (hesperidin and diosmin mixture) [248], and (i) inducing aggregation of SARS-CoV-2 spike proteins and disruption of the viral genome (hydroxytyrosol) [249]. Figure 2 shows the mechanisms of antioxidants compounds that could prevent neuronal damage in patients with COVID-19

## 6. Antioxidants as Neuroprotectors in Patients Infected with COVID-19

Given that nutrition and diet are closely linked to oxidative processes, they could also play an important role in the development and the severity of this disease and could impact in the progression of COVID-19 in patients. Plus, quarantine has been associated with depression and stress and a decrease in physical activity and nutrition quality in the general population [250]. Therefore, changes in lifestyle and diet must be implemented not only in the patients infected with COVID-19 but also in healthy people in order to decrease SARS-CoV-2 infection spread and lousy prognosis. As has been mentioned, no specific antiviral drug is available to treat COVID-19, but therapeutic interventions have helped to control COVID-19 in the world [251,252]. In this sense, it has been proposed that antioxidant compounds can improve the prognosis of COVID-19 patients through different mechanisms, mainly by the reduction of inflammation [252,253].

Modulation of the immune system by nutrition has been largely studied. Vitamins A, C, D, B6, and B12, folate, zinc, iron, copper, and selenium play a role in the immune response [152]. During COVID-19 disease, it has been shown that these micronutrients have a therapeutic effect. Vitamin C reduces inflammatory response, optimizes the immune system by modifying IL-6 and TNF-α levels, and maintains vascular consistency through its potent antioxidant properties [254]. Vitamin D has shown therapeutic effects in the viral infection by mechanisms such as the increase of reduced glutathione [255]. Vitamin D has shown a therapeutic role in reducing renin–angiotensin–aldosterone system activation as well as normalizing mitochondrial dynamics [256,257]. Recently, Gao and coworkers, in a retrospective cohort study, showed that in patients with COVID-19 infection with a high-dose of vitamin C (*n* = 46 patients, dose of 6 g intravenous infusion of vitamin C per 12 h on the first day, and 6 g once for the following 4 days) in comparison with standard therapy group (*n* = 30) the risk of the mortality was lower (HR = 9.91, 95% CI = 1.82–54; HR = 7.98, 95% CI = 1.24–51.22) in 28 days of therapy. The oxygen support status was also improved in patients with high-dose of vitamin C (63.9%) in comparison with patients in the standard therapy group (36.1%) over a median retrospective time of 18 days. In addition, the patients with a high dose of vitamin C had reduced serum high sensitivity-C-reactive protein, procalcitonin and IL-8 levels in comparison with the standard group. Finally the patients with a high dose of vitamin C did not show adverse effects [258]. In a retrospective case series study, vitamin C significantly decreased serum C-reactive protein from 0 to 3 after 7 days of administration in six patients infected with severe and six patients with critical COVID-19. The dosage of vitamin C applied in severe patients was 162.7 (71.1–328.6) mg/kg (body weight)/day and 178.6 (133.3–350.6) mg/kg/day in critical patients. The elevation of serum C-reactive protein was observed in both severe (59.01 ± 37.9 mg/mL) and critical (92.5 ± 41.21 mg/mL) and was decreased continuously at day 3 (12.36 ± 22.12 mg/mL) and day 7 (8.95 ± 20.4 mg/mL) in severe patients. For critical patients the dose was decreased at day 3 (33.9 ± 30.2 mg/mL) and slightly increased at day 7 (59.56 ± 41.4 mg/mL) [259].

Other antioxidant compounds that have been proposed as potential adjuvants in COVID-19 are zinc [260], selenium [261], melatonin [262], curcumin [263], N-acetylcystein [264,265], GC4419, a SOD mimetic [266], colchicine [267], α-lipoic acid [268], glutathione [269], broccoli or glucoraphanin capsules [270], dypiridamole [271], cannabidiol [272], combinations of vitamin D, magnesium, and vitamin B12 [273], and diammonium glycyrrhizinate with vitamin C [274].

Currently, some studies propose the use of antioxidants to treat neurological manifestations of COVID-19, such as melatonin. Melatonin is a neurohormone produced and secreted from the pineal gland; it is distributed to different brain structures by CSF and it has been reported to act as protectant for neurons and glia. In a recent review, Wongchitrat and coworkers suggested the use of melatonin supplementation against SARS-CoV-2 infection due to its antioxidants, anti-apoptotic, immunomodulatory, and anti-inflammatory effects. The melatonin anti-inflammatory effect has been related to the activation of different cellular pathways; Sirtulin-1 activation by melatonin inhibits macrophage polarization into pro-inflammatory phenotype. Melatonin also suppresses NF-kB activation, stimulates Nrf2 production, reduces pro-inflammatory cytokines (TNF-a, IL-1, IL-6, IL-8, IL-17), and increases levels of anti-inflammatory cytokines (IL-10). The therapeutic use of melatonin has been proposed for brain injury such as hypoxia/ischemia/stroke and during viral infections, and melatonin antiviral effects in vitro include a decrease in virus titer both in supernatant and cells. Studies in animal models demonstrate its impact in the reduction of NO and malondyaldehyde (MDA) levels as well as inducible nitric oxide synthase (iNOS) activity and apoptotic cell death during viral infections. Meningitis/encephalitis, Guillain–Barré syndrome, and hypoxic ischemia/stroke associated with SARS-CoV-2 are proposed to be treated with adequate levels of melatonin [262]. Cardinali and coworkers also propose the use of melatonin as an adjuvant for anti-SARS-CoV-2 infection treatment; the authors report that it can improve the cognitive decay observed in COVID-19 patients and during vaccination it could increase IgG antibody response and the number of CD8+ T cells [275].

Other antioxidant compounds proposed as potential neuroprotectants for handling neuronal injury and inflammation during COVID-19 are the phytochemicals resveratrol, quercetin, and kaempferol. Resveratrol has shown a capacity for inducing autophagy by AMPK/SIRT1 activation and PI3K/AKT/mTORC1/2 inhibition, which has been related to rescue of Nf2. Inhibition of immunoproteasome by resveratrol has also been associated with autophagy activation and prevention of PTEN degradation as well as the inhibition of NF-kB, Nod-like receptor pyrin containing (NLRPC) inflammasome, and pro-inflammatory cytokines, whose upregulation has been associated with brain injury during subarachnoid hemorrhage and neuroinflammation [276]. Quercetin and kaempferol, in addition to having antioxidant properties, are anti-inflammatory compounds acting by autophagy induction through NLRP3 inhibition and enhancement of IL-1 secretion mainly in microglial cells [277]. These effects results in diminution of oxidative stress and neuroinflammation, M2 microglia polarization, preservation of BBB function and activation of AMPK and Nrf2 in brain tissue.

Barré and coworkers proposed that Montelukast, a cysteinyl leukotriene receptor antagonist used as an anti-inflammatory agent in acute asthma, can be a potent neuroprotector in COVID-19 patients due to its capacity to limit BBB damage and anticonvulsant properties in epilepsy models as well as the improvement of recovery after brain ischemia through its effects on oligodendrocytes precursor cells. Plus, it has been demonstrated that Montelukast can reduce neuroinflammation and induce neurogenesis by GPR17 receptor. Several studies showed that Montelukast can mitigate cytokine storms and reduce interaction between SARS-CoV-2 and ACE2, as well as increase mitochondrial mass and function at systemic level [278].

HEMO_2_Life^®^ (M101) is an extracellular hemoglobin from the lugworm *Arenicola marina* with an oxygen capacity 40 times higher than the HbA of vertebrates, and it possesses anti-oxidative properties due to a superoxide dismutase activity-like based on copper and zinc. In animal models it showed an improvement of brain tissue oxygenation with HEMO_2_Life^®^ (M101). Lupon and coworkers proposed its use for hypoxemia and post-hypoxic leuko-encephalopathy presented in patients with COVID-19 [279].

In a screening of Ayurvedic products with the potential to modulate the immune system, Maurya and coworkers used the swissADME web tool to show that some molecules with antiviral, anti-inflammatory, and antioxidant properties such as nimbin, piperine, thebaine, berberine, andrographolide, zingiberene, citronellol, and eugenol have high affinity with the SARS-CoV-2 spike protein as well as good BBB diffusion, suggesting their potential use as neuroprotective agents in COVID-19 patients [280].

Koh and coworkers proposed the use of ergothioneine, a natural antioxidant found in non-yeast fungi and some bacteria, as a possible nutraceutical COVID-19-related neurologic complication. In some in vitro studies it has been shown that this compound is a powerful scavenger of hydroxyl radicals, hypochlorous acid, and peroxynitrite that can act as an antioxidant and cytoprotective and have a good penetration of the BBB. The authors also reported that ergothioneine protects against the damage induced by 7KC in brain endothelial cells [281,282].

Kempuraj and coworkers reviewed the neuroprotective effects in neurotrauma and neurodegeneration of flavone luteolin by inhibition of hydrogen peroxide, nitric oxide, and malondialdehyde and normalization of the activities of acetylcholinesterase, glutathione S-transferase, and superoxide dismutase and improvement of the neuroinflammatory responses in experimental models of neurotrauma. Additionally, some reports showed that luteolin can suppress neuroinflammatory response, activation of microglia and astrocytes, oxidative stress, neuroinflammation, and the severity of neuroinflammatory diseases such as Alzheimer’s disease, Parkinson’s disease, multiple sclerosis, and TBI pathogenesis. More recently it has been employed as a suppressor of neuroinflammatory response in COVID-19 patients, mainly because of its antiviral properties against SARS-CoV-2 by preventing the entry of the virus into the host cells and inhibition of TNF-α, IL-1β, IL-6, superoxide dismutase, and glutathione peroxidase, and its activation of microglia and astrocytes in neuroinflammatory conditions [283,284,285].

Ribeiro and collaborators proposed the use of P2X7 receptor antagonists for the prevention of neurological complications in COVID-19 patients. The hyperactivation of P2X7 receptors, ATP-gated ion channels expressed in the central nervous system, has been related to the inflammatory response induced by inflammasome activation and neuroimmune response activation, reactive oxygen species formation and glutamate release. The authors postulate that neuroinvasion of SARS-CoV-2 through the BBB observed in COVID-19 infection induces neuroinflammation mediated by hyperactivation of the P2X7 receptor and is associated with psychiatric disorders and neurodegeneration observed in this disease [286].

Uckun and collaborators proposed the use of Rejuveinix (RJX) as a neuroprotective agent in sepsis caused by COVID-19 infection. RJX is composed of natural antioxidants and anti-inflammatory agents that include ascorbic acid, magnesium sulfate heptahydrate, cyanocobalamin, thiamine hydrochloride, riboflavin 5′ phosphate, niacinamide, pyridoxine hydrochloride, and calcium D-pantothenate. The authors reported the capacity of RJX to improve the survival outcome of animals with lypopolisaccharide-galactosamine (LPS-GalN)-induced fatal sepsis caused by oxidative stress. The administration of RJX diminished the lipoperoxidation levels and normalized the levels of superoxide dismutase, catalase, and glutathione peroxidase in the mouse brain. According to these observations the authors propose the use of RJX in fatal sepsis and multi-organ failure observed in some patients infected with SARS-CoV-2 [287].

Other proposed candidates as neuroprotectors in patients with COVID-19 for their antioxidant capacity are: salicyl-carnosine [288], molecular hydrogen [224], cannabinoid type-2 receptor-selective agonists [289], propolis [290], thymoquinone, nigellidine, and α-hederin [291], agomelatine [292], and ozone [293].

Regarding the use of antioxidants for the treatment of neurological or psychiatric manifestations in COVID-19 patients to improve prognosis, Sher and coworkers showed that the use of melatonin improved a case of delirium related to COVID-19 in a case of a healthy woman aged 70, diagnosed with COVID-19 by PCR for SARS-CoV-2, treated with ceftriaxone, azithromycin, and remdesivir and intubated for hypoxic respiratory failure and extubated 26 days later. Three days after, the patient was diagnosed with delirium and treated with melatonin (15 mg per night) to regulate the sleep cycle and for its antioxidant and anti-inflammatory properties, concomitant with suvorexant (20 mg per night), guanfacine (0.5 mg from 0.5 mg per night titrated at 1 mg three times daily), valproic acid (1250 mg per day), quetiapine (initially titrated at 250 mg distributed throughout the day, but discontinued due to ineffectiveness), and haloperidol (8 mg per day). Although the patient had medical complications associated with lung damage from COVID-19, there was no evidence of delirium 10 days after treatment [161].

Olagnier and coworkers showed that SARS-CoV-2 infection induces the expression of genes related to inflammatory and antiviral pathways, including RIG-I receptor and Toll-like receptor signaling, in lung biopsies of COVID-19 patients as well as epithelial and kidney cell lines, and in genes associated to Nrf2 antioxidant response the expression is decreased. Using an in vitro approach, they explored the effects of the Nrf2 agonist dimethyl fumarate (DMF), a drug approved by the FDA for use as anti-inflammatory therapy for multiple sclerosis, and 4-octyl-itaconate (4-OI), a chemically synthesized, cell-permeable derivative of itaconate. Both compounds showed stimulation of antioxidant gene expression via Nrf2 induction in epithelial and kidney cell lines as well as primary human airway epithelial (HAE) cultures. Treatment with both agonists before SARS-CoV-2 cell infection reduced viral replication, the release of progeny, and cytotoxicity of SARS-CoV-2. The genetic activation of Nrf2 using siRNA silencing of KEAP1 (inhibitor of Nrfr2) led to the restriction of SARS-CoV-2 replication, confirming that DMF and 4-OI have antiviral properties by Nrf2 activation. The study showed that 4-OI had morphological activity without loss of cell viability. The activity of 4-OI did not seem to overlap with other compounds known to perturb cell morphology, including rapamycin, bafilomycin, tunicamycin, cyclohexamide, emetine, mitomycin, or doxorubicin. There was no observable overlap with the activity profile of remdesivir or hydroxychloroquine, indicating that the antiviral mode of action of 4-OI is distinct from other known antiviral mechanisms. The authors also demonstrate that 4-OI and DMF prevent inflammatory cytokine gene expression induced by SARS-CoV-2 such as *IFNΒ1*, C-X-C motif chemokine 10 (*CXCL10*), *TNFA*, *IL-1Β*, and C-C chemokine ligand 5 (*CCL5*) genes and increase Nrf2 inducible gene *HMOX1*. In addition, the effect of 4-OI on peripheral blood mononuclear cells (PBMC) from healthy donors and COVID-19 patients were examined. 4-OI treatment reduced *CXCL10* mRNA and reduced the induction of IFN and IFN genes in response to RIG-I agonist (M8), inhibiting interferon regulatory factor 3 (IRF3) dimerization. IRF3 inhibition has been associated with Nrf2 expression since its silencing is enough to restore IRF3 dimerization and limit the effect of 4-OI. The authors propose the use of DMF and 4-OI as Nrf2 agonists that can be used to inhibit SARS-CoV-2 replication as well as the expression of associated inflammatory genes in patients with COVID-19 [198].

Currently, the effect of several antioxidant compounds for the treatment of neurological or psychiatric manifestation in COVID-19 patients is under evaluation in clinical trials (see Table 3).

## 7. Perspectives

The current state of emergency due to the contingency derived from the COVID-19 pandemic has opened a wide range of questions, some of them with a response derived from the effort of the international scientific community, others in the process of being answered and others that will remain to be answered in the future. The reality of this disease caused by the SARS-CoV-2 virus has led us to understand that we are in the process of knowing both its clinical manifestations and its comorbidities, but above all its sequelae, the need to categorize depending on the age group, and the type of population affected, among so many other questions that make us think that this is only the beginning. We are in the first stages of understanding this disease since its worldwide recognition in December 2019, and therefore we need to keep monitoring COVID-19 to understand its implications and its effect on the chronicity of the symptoms and sequelae. This has led to a search for new therapeutic strategies that treat the acute symptoms of this disease and at the same time, we must consider how these strategies can impact the management of the sequelae. We must consider that this type of infection generates pro-inflammatory processes at the systemic and central level, which is why powerful pharmacological tools are required to address these alterations and their possible consequences, always taking into account avoiding unwanted, adverse effects.

We must point out that, although the damages generated at the systemic level are serious, at the neurological level they are not to be disregarded. The degrees of disability that can be generated are still in the process of being evaluated and the need to have therapeutic alternatives for the future is crucial. Treating and hypothesizing the possible mechanisms generated during this type of infection that can lead to these sequelae has become one of the main objectives in managing this pathology.

In particular, the use of antioxidant agents for the management of neurological diseases where an oxidative stress state occurs has been well received; this will allow us to transfer the knowledge and determine if these strategies can be used in the neurological alterations that are being reported and are attributed to SARS-CoV-2 infection. Currently, there is scientific evidence that these antioxidants can also act as anti-inflammatories which has led us to consider these therapeutic agents as having great potential for the management of this disease, considering that the most serious states of the disease are attributed to exacerbated pro-inflammatory states. This is in consideration of the mechanism known as cytokine storm, which has been reported in various age groups and in different populations, both with and without previous chronic diseases. 

It is worth mentioning that although different antioxidant substances that can be used for this purpose have been brought to light, the road is still long, and it is the task of the scientific community to continue with the studies in order to find the best therapeutic strategy for the management of COVID-19 without leaving behind the efforts in the research fields related to the generation of vaccines, as well as drugs with other characteristics that are focused in the treatment of symptoms, and comorbidities and sequelae both at a systemic and neurological level attributed to this type of infection.

## 8. Conclusions

It is imperative to continue with the study of the mechanisms that underlie CoV infections to gain understanding at different levels from genes to clinical practice; this will allow us to find the therapeutic targets that can be used for their management, particularly the substances that present antioxidant and anti-inflammatory activity which have already been reported to be effective in the treatment of other neurological diseases. Subsequent studies should be considered to establish the real effect both in the management of clinical symptoms and comorbidities, and on the sequelae (yet to be determined) derived from the damage presented by SARS-CoV-2 infection. Emphasis should be placed on establishing these measures according to the age group and the characteristics of the population affected by this disease.

## Figures and Tables

**Table 1:** Neurological manifestations in patients infected with COVID-19.

Type	Neurological Complications after COVID-19 Infection	Patients’ Origin	References
Inflammatory	Encephalitis Meningoencephalitis Cord myelopathy encephalitis Hypoxic encephalitis Autoinmune meningoencephalitis Acute-disseminated encephalomyelitis Autoimmune encephalitis Diffuse post hypoxic leukoencephalopathy Acute necrotizing encephalopathy Guillain–Barré Syndrome Guillain–Barré Syndrome associated with a cerebral vasculitis-like pattern Cerebillitis Mixed inflammatory cell Posterior reversible encephalopathy syndrome	Italy Iran United States Brazil United Kingdom India Egypt Mexico Canada Spain South Africa Netherlands Belgium France Peru Japan Germany Sweden	[62,76,77,78,79,80,81,82,83,84,85,86,87,88,89,90,91,92,93,94,95,96,97,98,99,100,101,102,103,104,105,106,107,108]
Vascular	Hemorrhage (intracerebral, subarachnoid, and intracranial) Multi-territory hemorrhagic infarctions Microbleeds masquerades Cerebral venous sinus thrombosis Embolic stroke in the right insula and left cerebellum Microinfarcts throughout the cortex Posterior cerebral artery infarct Middle cerebral artery territory infarcts Cuffing of intracerebral blood vessels distant from the infarcts Left cerebral small subdural hematoma with mild brain edema Vasculitis Perfusion abnormalities in brain Large vessel stroke Small subcortical infarcts Brain microvascular occlusive disorder Secondary acute ischemic stroke	United States South Africa Switzerland Germany Mexico India Saudi Arabia Brazil Japan Italy Spain China Turkey	[95,97,106,109,110,111,112,113,114,115,116,117,118,119,120,121,122,123,124,125,126]
Sensorial	Headache Vertigo Anosmia Ageusia Altered taste Migraine-like features Vision impairment Dizziness	Spain India Egypt China Canada Italy Turkey Germany United States Venezuela Bolivia	[72,97,98,127,128,129,130,131,132]
Behavioral	Confusion Seizure Convulsions Cognitive decay coma Neuropsychiatric disorder Delirium Maniac-like symptoms Depression Altered mental status Psychosis Dementia-like syndrome Dysexecutive syndrome	France China Iran Egypt Saudi Arabia Belgium Spain India United Kingdom	[69,72,96,97,98,125,133,134,135,136,137,138]
Peripheral	Peripheral neuropathy Myasthenia gravis Symmetric hypokinetic-rigid syndrome Cranial neuropathy Nerve pain Bell’s palsy Balint–Holmes’ syndrome Ataxia Anti-diuretic hormone secretion	Belgium Egypt Spain China India Italy	[72,97,98,99,135,139,140]
Anatomical lesions	Transtentorial herniation Cytotoxic lesions of the corpus callosum Diffuse corticospinal tract Brain and spine demyelinating lesions Pneumocephalus	United States Italy Saudi Arabia France	[69,125,126,141,142,143,144]

**Table 2:** Treatment protocols for neurological or psychiatric manifestations induced by COVID-19.

Type	Neurological Condition	Treatment Protocol	Reference
Immunological	▪ Encephalitis	○ Lopinavir/ritonavir (400/100 mg), subcutaneous interferon β-1B (250 μg/48 h), intravenous immunoglobulins (0.4 g/kg/day/5 days), and intravenous tocilizumab (400 mg/24 h/3 days).	[83]
		○ Lopinavir/ritonavir, hydroxychloroquine sulfate (200 mg twice/day), and antibiotics (piperacillin/tazobactam).	[99]
	▪ Guillain–Barré Syndrome	○ Intravenous immunoglobulins ○ Tacrolimus (2 mg/twice a day), mycophenolate (540 mg/twice a day), prednisone (5 mg/day), intravenous immunoglobulin (0.4 mg/kg/day/5 days)	[62,101]
	▪ Meningoencephalitis	○ Intravenous immunoglobulins therapy (0.4 g/kg)	[164]
	▪ Autoimmune encephalitis	○ Remdesevir (200 mg on the first day followed by 100 mg/daily/4 days) and dexamethasone (6 mg/10 days), valproic acid (1500 mg/day), intravenous methylprednisolone (500 mg/daily/5 days, 250 mg/day/3 days, 125 mg/day/3 days) followed by oral prednisolone gradually reduced	[92]
		○ Immunoglobulins (0.4 g/kg/day/5 days), methylprednisolone (1 g/day/5 days), and clonazepam (0.3 mg three times a day)	[93]
	▪ Acute necrotizing encephalopathy	○ Intravenous methylprednisolone (5 days)	[100]
	▪ Acute disseminated encephalomyelitis	○ Intravenous methylprednisolone (1 g/3 days) followed by a weaning course of prednisolone, starting at 60 mg/day and reducing by 10 mg/week until on a maintenance dose of 20 mg	[86]
	▪ Meningitis	○ Ceftriaxone, azithromycin, and hydroxychloroquine	[94]
	▪ Posterior reversible encephalopathy	○ Antiviral therapy with darunavir/cobicistat, associated with hydroxychloroquine	[108]
	▪ Brain and spine demyelinating lesions	○ Antiretroviral and hydroxychloroquine; antiepileptic therapy with lacosamide, levetiracetam, and phenytoin; dexamethasone (20 mg/day/10 days)	[144]
Anatomical	▪ Cytotoxic lesions of the corpus callosum	○ Dexamethasone, favipiravir, piperacillin tazobactam, and azithromycin	[143]
Behavioral or Psychiatric	▪ Maniac-like symptoms	○ Arbidol Tablets, Moxifloxacin, Darunavir and Cobicistat Tablets, and methylprednisolone intramuscular injection (5–2.5 mg haloperidol twice/day) on the first 2 days of the maniac-like attack, combined with olanzapine which was gradually titrated to 10 mg/day	[99]
	▪ Delirium	○ 10-day course of oral hydroxychloroquine therapy (200 mg/8 h/10 days) as well as a course of intravenous piperacillin/tazobactam (1 g/8 h/7 days)	[136]
	▪ Acute hypokinetic-rigid syndrome	○ Hydroxychloroquine (200 mg/12 h/10 days), lopinavir/ritonavir (400/100 mg/12 h/12 days), interferon β (0.25 mg/6 days), ceftriaxone (2 g/24 h/10 days), Tocilizumab (680 mg/1 day), azithromycin (500 mg/24 h/5 days), methylprednisolone (250 mg/24 h/3 days), and levetiracetam (1000 mg/12 h/4 days)	[139]
Vascular	▪ Secondary acute ischemic stroke	○ Atorvastatin (20 mg, p.o., q.d.), tirofiban (0.1 μg/kg.min, continuous intravenous pumping for 48 h), daily aspirin (100 mg, p.o.), and clopidogrel (75 mg, p.o.)	[110]
	▪ Brain microvascular occlusive disorder	○ Steroids, hydroxychloroquine, and ceftaroline with a prophylactic dose of enoxaparin (6000 U once/day)	[116]
	▪ Subarachnoid hemorrhage	○ Heparin infusion, tPA (2.0 mg/h)	[120]
	▪ Large-vessel stroke	○ Apixaban, aspirin, tPA and clopidogrel	[113]
	▪ Cerebral venous sinus thrombosis	○ Enoxaparin anticoagulation treatment and prophylactic Levetiracetam infusion	[125]

**Table 3:** Current clinical trials evaluating the effect of antioxidant compounds on neurological manifestations in COVID-19.

Antioxidant(s)	Protocol	Neurological or Psychiatric Conditions Assessed
Vitamin C	▪ Bolus doses of 50 mg/kg in 50-mL of either dextrose 5% in water or normal saline for 30 to 60 min, every 6 h for 96 h (200 mg/kg/day and 16 doses in total) (N = 800, protocol in recruiting)	○ Pain, discomfort, anxiety/depression, mobility (by 6 months) and consciousness (up to 28 days)
Zinc and vitamin C	▪ Standard treatment + 220 mg (zinc) and 1 g (vitamin C)/24 h/10 days (N = 50, protocol not yet recruiting)	○ Muscle or body aches, headache, loss of taste, loss of smell by 28 days
	▪ Standard treatment + 8000 mg (vitamin C) divided into 2–3 doses/day with food and 50 mg (zinc gluconate) daily at bedtime (N = 520, protocol in recruiting)	○ Headache, loss of taste, loss of smell by 28 days
	▪ 80 mg (zinc)/500 mg (vitamin C) daily for 42 days additional to hydroxychloroquine, ivermectin, and povidone iodine treatment (N = 4257, protocol completed)	○ Change in smell by 42 days
	▪ Standard treatment + 8000 mg (zinc) divided into 2–3 doses/day with food and 50 mg (zinc gluconate) daily at bedtime (N = 214, protocol with recruitment completed)	○ Muscle and body aches, headache, new loss of taste and new loss of smell by 28 days
Hydroxychloroquine, vitamin C, vitamin D, and zinc	▪ Treatment with hydroxychloroquine will last 1 day. Treatment with vitamin C, vitamin D, and zinc will last 12 weeks (N = 600, protocol in recruiting)	○ Headache and difficulty speaking by 24 weeks
Melatonin	▪ Oral 10 mg/three times a day for 14 days (N = 30, protocol in recruiting)	○ Headache, loss of taste, loss of smell and dizziness by 28 days
Glycine and N-acetylcysteine	▪ Supplementation by 2 weeks (N = 64, protocol in recruiting)	○ Change in cognition and memory assessed up to 10 weeks
Glycine	▪ Habitual treatment + 0.5 g/kg/day by nasogastric tube/4 equal doses per day (N = 82, protocol in recruiting)	○ Consciousness assessed up to 12 months
Vitamin C, vitamin E, melatonin, and N-acetylcysteine	▪ One antioxidant plus pentoxifylline. Vitamin C 1 g/12 h. Vitamin E 800 mg/24 h. Melatonin 50 mg/24 h. N-acetylcysteine 600 mg/12 h (N = 11, protocol active not recruiting)	○ Consciousness for 7 days post antioxidant dose
Previfenon^®^	▪ Contains 250 mg of epigallocatechin-3-gallate (EGCG). Total EGCG dose per patient will be 750 mg/day (3 capsules/8 h) for 40 consecutive days (variable time between 60 and 70 days) (N = 524, protocol in recruiting)	○ Myalgia, loss of smell, loss of taste, headache by 40–70 days of intervention
Hesperidin and diosmin	▪ 1000 mg/8 h for 7 days and 1000 mg/12 h times daily for 3 days (N = 100, protocol not yet recruiting)	○ Myalgia by 14 days
Fuzheng Huayu Tablet (FZHY) and vitamin C	▪ FZHY tablet administration: 0.4 g/tablet, 1.6 g/time, three times/day, medicine to be taken after meals. Vitamin C tablets administration: 0.2 g/time, three times/day. (N = 160, protocol in recruiting)	○ Insomnia by 24 weeks
Resveratrol	▪ Capsules of 1.0 g, orally once a day for six months. (N = 30, protocol not yet in recruiting)	○ Depression by 9 months

www.clinicaltrials.gov, accessed on 22 April 2021 [294].
